# Parametric Study of Bolt Clamping Effect on Resonance Characteristics of Langevin Transducers with Lumped Circuit Models

**DOI:** 10.3390/s20071952

**Published:** 2020-03-31

**Authors:** Jinhyuk Kim, Jungwoo Lee

**Affiliations:** Department of Electronic Engineering, Kwangwoon University, Seoul 01897, Korea; 2018160201@kw.ac.kr

**Keywords:** Langevin transducer, Mason’s model, T-circuit model, resonance characteristics, effective electromechanical coupling coefficient

## Abstract

We recently proposed a numerical model using equivalent circuit models to analyze the resonance characteristics of Langevin transducers and design them in a systematic manner. However, no pre-load torque biased by a metal bolt was considered in the model. Here, a parametric study is, therefore, carried out to reveal how model parameters are adapted to incorporate the pre-compression effect into our existing model. Analytical results are compared with corresponding experimental data, particularly regarding the input electrical impedance and effective electromechanical coupling coefficient for the transducer at resonance modes. The frequency response of input impedance is presented as a function of torque, both theoretically and experimentally. For 10.0 N·m bias, for instance, both resonance and anti-resonance frequencies are calculated as 38.64 kHz and 39.78 kHz, while these are measured as 38.62 kHz and 39.77 kHz by the impedance analyzer. The impedance difference between these cases is 14 Ω at resonance and 9 kΩ at anti-resonance, while the coupling coefficients in both cases become 0.238 and 0.239, respectively. Hence, these test results are closely matched with their theoretical values. Consequently, this study provides a quantitative guideline that specifies the pre-loading condition of bolt clamps with proper parameter settings to predict the intended resonance characteristics of Langevin transducers.

## 1. Introduction

Bolt-clamped transducers, also known as Langevin transducers, are widely used, giving rise to a boosted ultrasound transmission for various industrial or medical applications including welding [1], cleaning [2], and liposuction [3]. In many cases, mechanical energy carried by acoustic waves in a frequency range of tens of kHz often disrupts the molecular integrity of metal or fatty structures by interrogating a highly focused ultrasound beam at their specified areas. Several analytical studies were, thus, carried out to propose design methods of Langevin transducers for such uses [4,5,6].

The typical transducer structure is composed of piezoelectric layers, front/back masses, a horn, and a metal bolt. A pre-stressed central bolt clamps one or more pairs of piezo-rings, particularly made of lead zirconate titanate (PZT), with other acoustically passive elements in order to apply mechanical bias to the whole transducer. Recently, we devised a systematic design approach based on equivalent circuit models to examine the resonance characteristics of therapeutic Langevin transducers [7]. In that study, Mason’s model consisting of two mechanical ports and one electrical port separated through an ideal transformer was used to describe the complex behavior of piezoelectric ceramics, while the T-network model was used to represent other passive parts of the transducer. Design variables were inherent material properties of each element, e.g. their physical dimension, acoustic impedance, dielectric constant, etc., Simulation results regarding input impedance and electromechanical coupling coefficient were presented under various conditions, demonstrating that our model was capable of specifying the desired resonance feature within an acceptable margin.

Although the piezoelectric layer is sandwiched between front and back masses by a bolt of high tensile strength, little attention was paid to the proposed model to accommodate pre-compressive bias or the pre-load applied by the bolt. In order to withstand higher loads and guarantee effective energy transfer, the piezoelement needs to be placed under a compressive stress through the pre-loading process. In this paper, therefore, the loading effect of the bolt clamp is further investigated with our aforementioned model. Since the pre-loaded stress may give rise to changes in electrical quantities, the intrinsic properties of piezo-layers, such as sound speed, are varied to calculate the magnitude of input electrical impedance as a function of frequency. Resonance and anti-resonance frequencies are identified from the impedance curve, and its effective electromechanical coupling coefficient is subsequently estimated. A comparison between simulation and experimental results is undertaken, and a relevant discussion is presented in the following sections.

## 2. Materials and Methods

### 2.1. Structure of Langevin Transducers

**Figure 1:**
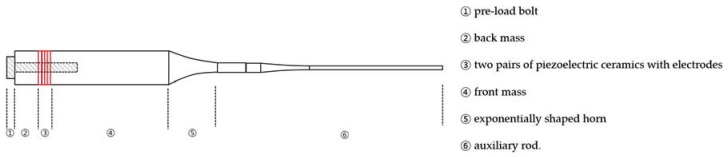
Structure of typical Langevin transducers.

Typical Langevin transducers comprise a piezoelectric stack, metal mass, central bolt, and ultrasonic horn booster, as illustrated in Figure 1. The piezoelectric layer is sandwiched between back and front masses which are clamped together by the bolt, and the transducer parts are entirely mechanically assembled in series.

The diagonally lined area represents the pre-loaded bolt that goes through the back mass, piezoelectric layer, and front mass at once. Stainless steel of high fatigue strength and low mechanical loss is most commonly preferred for the bolt material. One or more PZT-4 pairs are used to fabricate high-power transducers since PZT has a high mechanical Q-factor and low mechanical and dielectric losses. The back mass reflects longitudinal waves from piezoelectric elements in the forward direction, and the appropriate material for this is also stainless steel due to its high acoustic impedance and mass density. The front mass, such as aluminum, facilitates forward acoustic energy transmission and is required to meet lower acoustic impedance than that of piezoelectric layers. The horn-shaped element serves as a power booster to amplify the acoustic energy and may have various auxiliary tools attached—e.g., a welding tool and surgical instrument—to the front end. 

In order to specify the longitudinal dimension of a Langevin transducer, the half-wavelength principle is exploited to determine each length of the transducer part that requires integer multiples of half wavelength *λ_r_*/2 at resonance frequency *f_r_*. This constraint is employed to make sure of the free stress boundary condition at both ends of each part. Individual elements are then assembled and finally form a complete resonating device [8]. 

### 2.2. Metal Bolt vs. Electromechanical Coupling Coefficient

The piezoelectric tensile stress needs to be reinforced to maximize the magnitude of longitudinal oscillation by providing piezoelectric elements with consistent compressive torque. In this regard, a metal bolt plays a crucial role in clamping through the front and back masses as it prevents the transducer from operation failures, such as depolarization and the structural distortion of piezoelectric materials. Proper pre-load torque maintains the solid contact of each interface between the acoustic stacks, and, in turn, longitudinal pressure transmits throughout the transducer with minimal mechanical loss. However, the piezoelectrical constant is decreased when the compressive stress becomes excessive [9]. Such a decrease may adversely affect both the polarization and mechanical loss of piezo-layers, leading to unexpected temperature surges [10].

Along with the conversion ratio between the mechanical and electrical energy applied to or from the piezoelectric element in thickness mode, the electromechanical coupling coefficient *k_t_* is expressed in Equation (1), where *f_r_* is the resonance frequency and *f_a_* is the anti-resonance frequency of a piezoelectric part. Other studies already showed that such a compression of the piezoelement by a stiff metal component may influence the transducer’s resonance feature, with *k_t_* being the result [11,12].

(1) $$k_{t}=\sqrt{\left(\frac{\pi }{2}⦁\frac{f_{r}}{f_{a}})\cot(\frac{\pi }{2}⦁\frac{f_{r}}{f_{a}}\right)}$$

In addition, the clamped capacitance *C*_0_ of a piezo-material is dependent on *k_t_*, as expressed in Equation (2). *A* is the area, *ε*_0_ is the relative dielectric constant, *ε_r_* is the relative dielectric constant, *L* is the length, and *n* is the number of piezoelectric layers.

(2) $$C_{0}=\frac{\left(n⦁A⦁\varepsilon_{0}⦁\varepsilon_{r}\right)\left(1-k_{t}^{2}\right)}{L}$$

In practice, the effective coupling coefficient *k_eff_* is used with Equation (3) to evaluate the sensitivity of the transducer.

(3) $$k_{eff}=\sqrt{1-\frac{f_{r}^{2}}{f_{a}^{2}}}$$

### 2.3. Equivalent Circuit Model

A numerical model is formulated to calculate the electrical input impedance of Langevin transducers over a certain frequency range. There are various equivalent models for representing the complex behavior of the piezoelectric material including Krimholtz, Leedom, and Matthae (KLM), Mason, Van Dyke, and Leach models [13,14,15]. Among them, the Mason model exhibits simpler electrical analogy than the others, consisting of two terminal impedances, a cross-impedance, and a clamped capacitance separated through an ideal transformer. Passive components including front/back masses and booster horns are then described with a T-shaped equivalent circuit [16]. This T-circuit model is capable of considering a variety of cross-sections and, therefore, the effect of curved profiles such as exponentially shaped horns can be reflected by the electrical impedance of the transducer. Finally, these two lumped circuits are connected in series to form a complete analytical model of the transducer. More details on our analytical model can be found in Reference [7].

### 2.4. Fabrication of Langevin Transducers

**Figure 2:**
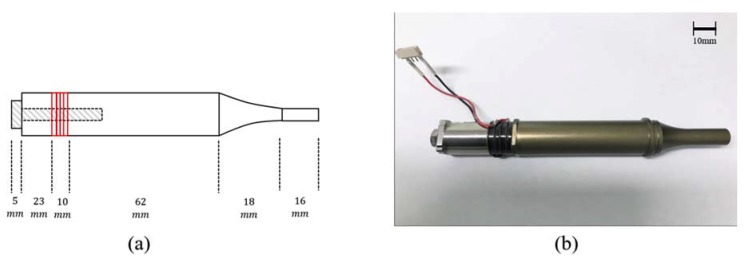
Transducer configuration used in this study: (**a**) simplified diagram; (**b**) actual transducer.

The transducer architecture is given in Figure 2a, where the length of each component is indicated. Lathe work is done to process cylinder-shaped stainless steel with a hole for the insertion of a pre-loading bolt. The number of piezoelectric elements used in the design depends on the application of the transducer, target resonance frequency, and electrical loading condition [17]. As in typical medical devices [18], four PZT-4 (c-203, Fuji Ceramic Corporation, Fujinomiya, Japan) rings are positioned between two metal masses. Each thickness of 2.5 mm is determined to meet the constraint of the half-wavelength rule at 38 kHz. To excite the transducer with a sinusoidal signal, 0.1-mm copper–nickel plates are inserted between adjacent PZT-4 layers, where the plate thickness is considered negligible in our model. Electrical contact is set by wires attached to the side of PZT-4 layers with conductive paste.

For efficient transmission, aluminum is employed as the front mass due to its durability, ease of manufacture, and lower acoustic impedance than PZT-4. Both back and front masses are electrically grounded via a black lead line, as shown in Figure 2b. There are a variety of booster shapes, including stepped horn, conical horn, Gaussian-curved horn, and so on. We use an exponentially shaped horn here because it allows structural stability, a proper amplification ratio, and design simplicity through lumped circuit models [19]. The decaying rate at which the horn’s cross-sectional area decreases is 34.7 m^−1^ in the axial direction. Front/back masses and PZT-4 stacks are clamped at once with stainless steel bolt through a center hole. In general, part of the transducer including the front mass and horn is immersed in liquid media during operation, and the corrosion resistance needs to be enhanced by proceeding with the anodizing treatment on the front surface. Finally, the individual material properties and physical dimensions for each transducer part are summarized in Table 1.

## 3. Results

Based on the equivalent circuit model derived so far, a MATLAB simulation was carried out to calculate the electrical input impedance with a target resonance frequency at 38 kHz. To verify our analytical results, the magnitude of the input impedance was measured with an impedance analyzer (4294A, Agilent, Santa Clara, CA, USA), while the pre-loaded torque ranging from 6.0 N·m to 10.0 N·m was produced with increment of 0.5 N·m by a digital torque-wrench (WPC3-030, Bluetec, Taichung, Taiwan). Here, it was observed that the torque increment led to a nonlinear change in the strain–stress relationship of PZT-4 and, in turn, the Young’s modulus. The tangential slope of the strain–stress curve varied from 5.16 to 5.99 × 10^10^ N/m^2^, depending on the applied torque. Sound speed in materials—one of our model parameters—explicitly influences other parameters such as terminal and cross-impedances in the Mason model and T-circuits. Note that the square of the sound speed is proportional to the modulus. Under the saturation of Young’s modulus toward stronger torque, the sound speed in PZT-4 was, thus, adjusted from 2591 m/s to 2791 m/s and linearly increased by 25 m/s for the sake of simplicity. Each torque–resonance test was repeated twice to provide statistical measurements. The number of test rounds was carefully chosen, since the excessive repetition of a compression–release procedure leads to metal fatigue, which adversely affects the mechanical integrity of the transducer. A one-hour aging period after bolt clamping is necessary to make sure that the torque fully settles throughout the transducer.

**Figure 3:**
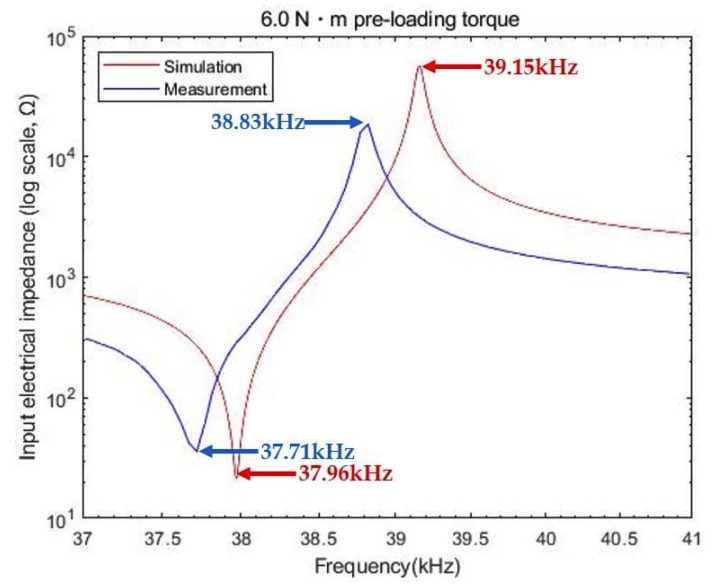
Simulation vs. test result of input impedance for 6.0 N·m torque.

The impedance curves from simulation and measurement data are depicted in Figure 3. *f_r_* and *f_a_* were computed at 37.96 kHz and 39.15 kHz in simulation, where their corresponding impedances were 23 Ω and 55 kΩ, respectively. The resultant *k_eff_* was then 0.245. According to the impedance analyzer test, in contrast, these frequencies were found at 37.71 kHz and 38.83 kHz, respectively, where the measured average *k_eff_* was 0.236. The measured impedances were 35 Ω and 18 kΩ. The difference in *k_eff_* between the model and experimental results was 2.5%.

However, a relatively marked discrepancy in both the resonance location and the impedance was observed under weak clamping condition. This may occur when a resonance component is unexpectedly introduced to the PZT-4 stack due to insufficient compression. An earlier work showed that additional anti-resonance can reduce the overall frequency response of vibration by the equivalently cascading supplementary series impedance, which represents the inertial and elastic lumped elements with the sandwiched stack [23].

**Figure 4:**
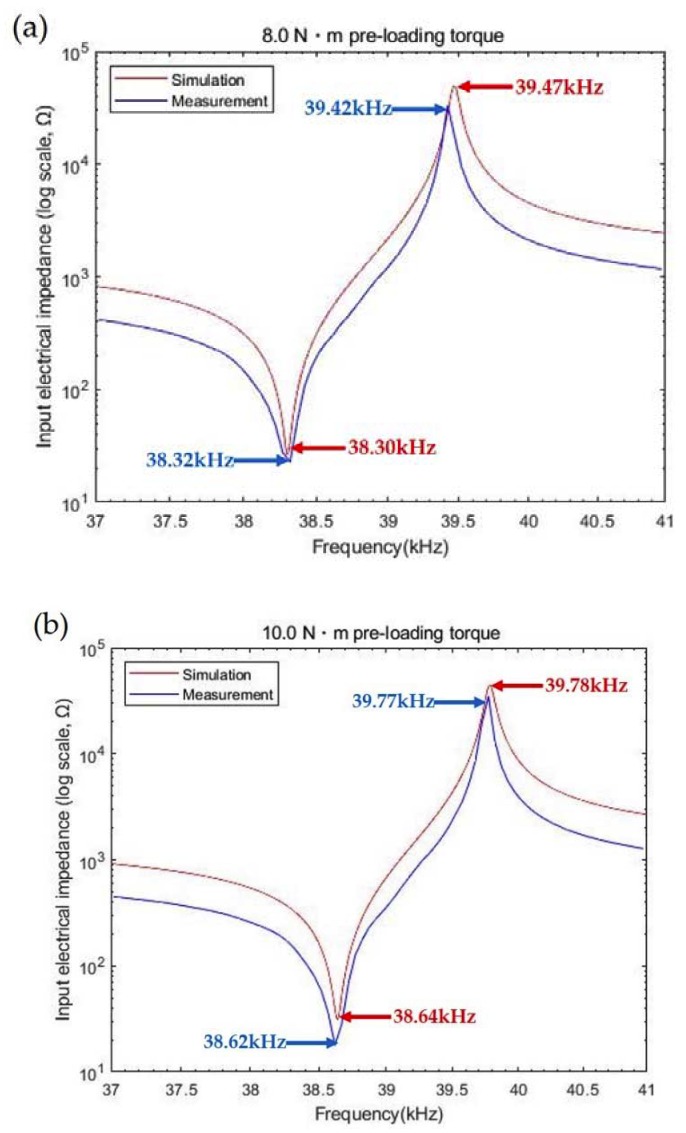
Comparison between simulation and test results for (**a**) 8.0 N·m and (**b**) 10.0 N·m.

Similarly, the pre-loading effect of the bolt clamp on resonance characteristics is further examined in 8.0 N·m and 10.0 N·m cases. For 8.0 N∙m compression in Figure 4a, *f_r_*, *f_a_*, and *k_eff_* were 38.30 kHz, 39.47 kHz, and 0.242 in the model estimation, whereas these values were 38.32 kHz, 39.42 kHz, and 0.235 in the equipment test. The difference in *k_eff_* was 2.9%. Input impedances were 27 Ω and 22 Ω at resonance, and 49 kΩ and 33 kΩ at anti-resonance. Impedance differences at these resonance peaks were, thus, 5 Ω and 16 kΩ, respectively. For 10.0 N·m stress, in addition, the same dataset was obtained in Figure 4b as 38.64 kHz, 39.78 kHz, and 0.238 for computation, while these values were 38.62 kHz, 39.77 kHz, and 0.239 for measurement. The error in *k_eff_* was 0.4%. Impedances for simulation and test were 31 Ω and 17 Ω at resonance, and 43 kΩ and 34 kΩ at anti-resonance. The impedance differences in each resonance mode were then 14 Ω and 9 kΩ. Hence, the pre-loading condition was less correlated with *k_eff_* than with impedance change as higher torque was applied.

The minimum impedance difference was achieved for 8 N·m at resonance and was suitable for driving the transducer with peripheral instruments which were electrically matched at 50 Ω. As the torque increased from 6 N·m to 10 N·m, the impedance discrepancy at anti-resonance rapidly dropped from 37 kΩ to 9 kΩ. This can be justified since series impedance terms that may be added to existing piezoelectric impedance are more likely to be neglected under stronger torque, as discussed in Reference [23].

**Figure 5:**
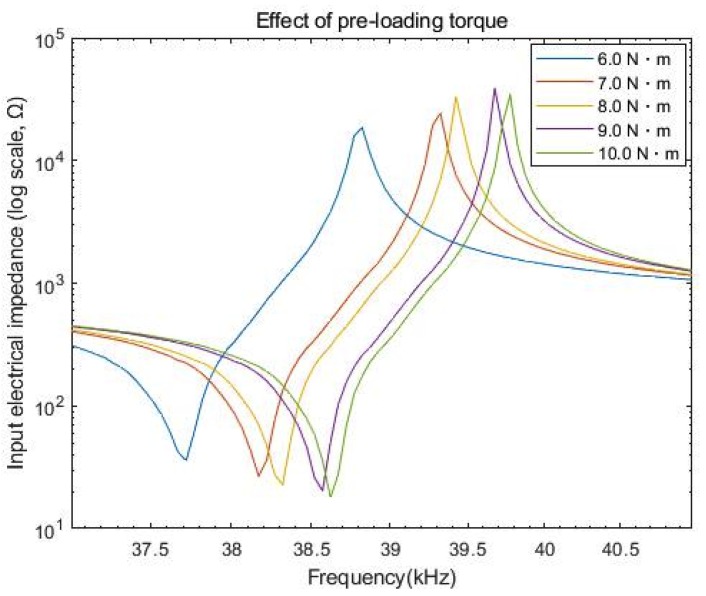
Frequency response of impedance as a function of pre-loading torque.

**Figure 6:**
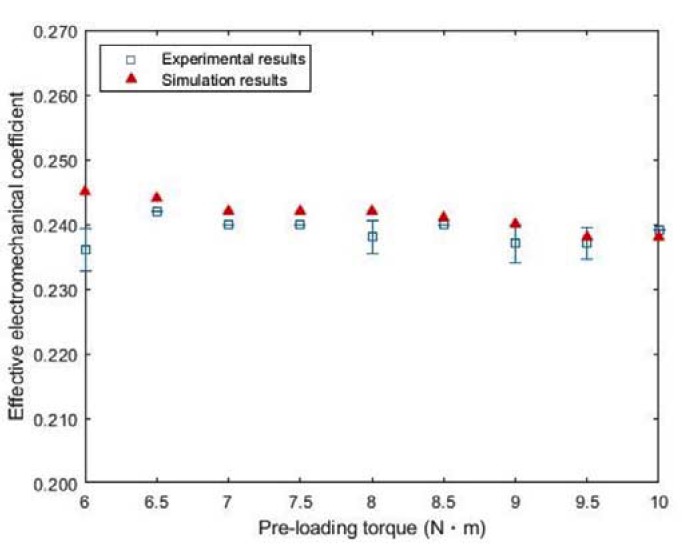
Simulation and experimental results regarding *k_eff_* as a function of pre-load torque.

The measured impedance spectrum is plotted in Figure 5 as a function of pre-loading torque. When the torque changed from 6.0 N·m to 10.0 N·m, the magnitude of the impedance at resonance reduced from 36 Ω to 18 Ω, while it increased from 19 kΩ to 40 kΩ at anti-resonance. Such distinct impedance characteristics can be explained by the presence of parasitic capacitances [24], which are considered to be connected to the piezoelement in parallel at resonance and in series at anti-resonance. These capacitive terms may have nonlinear dependence on the torque, as resulting from multiple capacitors formed by pairs of PZT-4 and electroplate. By adding these extra impedance terms, as a result, the total equivalent impedance of PZT-4 can decrease at resonance and conversely increase at anti-resonance. Both model estimates and experimental data regarding *k_eff_* deviated within a narrow margin, as represented in Figure 6. In particular, the analytical results indicate that the maximum *k_eff_* was 0.245 at 6.0 N∙m, and its minimum value was 0.238 for 9.5 N·m and 10 N·m. Meanwhile, the measured *k_eff_* reached its maximum value at 0.242 for 6.5 N·m, and the minimum value was 0.236 for 6.0 N·m. Since, however, *k_eff_* is computed with Equation (3) assuming a linear regime, our model may not exactly reflect the nonlinear resonance caused by compressive force. In fact, the *k_eff_* value for simulation was greater than its measured result only when the applied torque was below 8 N·m. A slight decrease in *k_eff_* beyond 8.5 N·m may have arisen from the depolarization of PZT-4 induced due to increased pressure on its surface [25].

As the bolt tightened, a frequency shift in resonance behavior noticeably occurs toward higher frequency, but the variation of *k_eff_* was less apparent in test results. From 7.0 N·m to 8.5 N·m, for example, *k_eff_* remained constant at 0.240 except for the value of 0.235 for 8.0 N·m in Table 2. In contrast, a similar experimental trend was seen in the same torque range, showing in Table 3 that *k_eff_* was determined to be 0.242 except for the value of 0.241 in 8.5 N·m. This finding implies that it is possible for us to select a target resonance band without considerably compromising the transducer sensitivity.

## 4. Conclusions

This paper investigated the effect of mechanical bias on the resonance characteristics of Langevin transducers by comparing numerical estimates with experimental results. Both the Mason and T-shaped equivalent circuit models were employed to analyze the magnitude of input electrical impedance over a given torque range. It was assumed that bolt clamping gave rise to the change in the elastic property of transducers, which enabled us to vary the sound speed of piezoceramics in our theoretical model. As a function of pre-loaded torque by metal bolts, the impedance spectrum was acquired both numerically and experimentally along with its electromechanical coupling coefficient. This parametric study proposes an effective way to include a compressive stress factor in transducer design in association with physical size, material property, resonance bandwidth, and so on. The results obtained here do not fully justify the complex resonance behavior of transducers, since many parameters in our model are linked with each other and there is a restriction to assessing their collective behavior by using any single factor. However, this lumped-circuit based approach can still provide approximate solutions to the stress-involved resonance problem in a more simplified manner by selecting the most explicitly affected factor as a variable. In subsequent works, stress- or temperature-dependent parameters such as the piezoelectric constant and dielectric permittivity [26,27] may be incorporated into our existing model by supplementing relevant lumped elements to evaluate nonlinear piezoelectric characteristics.

## Figures and Tables

**Table 1:** Material properties by transducer parts.

Part	Material	Sound Speed (m/s)	Density (kg/m^3^)	Acoustic Impedance (Mrayls)	Outer Diameter (mm)	Inner Diameter (mm)
Metal bolt	Stainless steel [20]	5920	8000	47	20	
Back mass	Stainless steel	5920	8000	47	20	6.5
Piezoceramics	Hard ceramic [21]	2791	7700	21	20	6.5
Front mass	Aluminum [22]	6375	2700	17	20	6.5
Horn	Aluminum	6375	2700	17	20	

**Table 2:** Simulation results in the presence of pre-loading torque.

	6.0 N∙m	6.5 N∙m	7.0 N∙m	7.5 N∙m	8.0 N∙m	8.5 N∙m	9.0 N∙m	9.5 N∙m	10.0 N∙m
*f_r_* (kHz)	37.96	38.05	38.13	38.22	38.30	38.38	38.46	38.56	38.64
*f_a_* (kHz)	39.15	39.24	39.30	39.39	39.47	39.55	39.62	39.70	39.78
*k_eff_*	0.245	0.244	0.242	0.242	0.242	0.241	0.240	0.238	0.238

**Table 3:** Measurement results under pre-loading condition.

	6.0 N∙m	6.5 N∙m	7.0 N∙m	7.5 N∙m	8.0 N∙m	8.5 N∙m	9.0 N∙m	9.5 N∙m	10.0 N∙m
*f_r_* (kHz)	37.71	38.07	38.17	38.22	38.32	38.37	38.57	38.57	38.62
*f_a_* (kHz)	38.83	39.17	39.32	39.37	39.42	39.52	39.67	39.72	39.77
*k_eff_	0.236 (0.003)	0.242 (0)	0.240 (0)	0.240 (0)	0.238 (0.002)	0.240 (0)	0.237 (0.003)	0.237 (0.002)	0.239 (0)

* Note that each averaged *k_eff_* is shown with its standard deviation in parentheses.
